# Design and Ultra-Precision Fabrication of Freeform Fresnel Lenses for Generating Rectangular Dark Hollow Beams

**DOI:** 10.3390/mi17040448

**Published:** 2026-04-03

**Authors:** Juan Zhang, Qilu Huang, Yingxin Xu, Chaocheng Yang, Tingdi Liao

**Affiliations:** 1 Institute for Photonics Technology, Quanzhou Normal University, Quanzhou 362000, China; 2 College of Photonic and Electronic Information Engineering, Fujian Normal University, Fuzhou 350000, China; 3 Fujian Provincial Collaborative Innovation Center for Ultra-Precision Optical Engineering and Applications, Quanzhou 362000, China; 4 Fujian Provincial Key Laboratory for Advanced Micro-Nano Photonics Technology and Devices, Quanzhou 362000, China

**Keywords:** freeform Fresnel lens, rectangular dark hollow beam, beam shaping, ultra-precision diamond turning

## Abstract

Freeform Fresnel lenses combine the powerful beam-shaping capability of freeform optics with the lightweight and compact characteristics of conventional Fresnel structures, leading to their increasing adoption across diverse applications. This paper proposes and experimentally validates a method for generating rectangular dark hollow beams using a freeform Fresnel lens. The lens is divided into multiple fan-shaped sectors centered on the optical axis, with each sector generating a defocused spot at a distinct spatial location. Based on geometrical optics, a freeform Fresnel lens with a 25 mm aperture is designed to produce a square hollow beam with a side length of 10 mm. A lens with a division angle of 5° was fabricated using ultra-precision diamond turning. The angular form error was measured to be below 0.1°, and the surface roughness was found to be below 10 nm. An optical testing system was established to characterize the generated beam profile. The experimental results successfully demonstrate the formation of the desired rectangular dark hollow beam. The measured results agree well with the simulations, confirming the feasibility and practical potential of the proposed method.

## 1. Introduction

Dark hollow beams (DHBs) are structured optical fields characterized by low on-axis intensity and a surrounding high-intensity boundary. They have been widely studied in atom optics [1,2], optical communications [3], laser material processing [4], and optical manipulation of particles [5,6]. Most existing studies focus on DHBs with circular symmetry. However, several theoretical models for DHBs with non-circular symmetry have also been reported [7,8,9,10]. Elliptical hollow beams can be used to trap microscopic particles and guide them along elliptical trajectories. They can also trap and rotate single elongated objects [11]. In addition, under certain conditions in weak atmospheric turbulence, rectangular DHBs exhibit a lower scintillation index than Gaussian, elliptical Gaussian, and rectangular flat-topped beams [12]. Freeform DHBs are particularly suitable for manipulating non-spherical objects in optical tweezers, as they help suppress unwanted rotational motion of trapped micro-objects [13].

Various methods have been proposed to generate DHBs, including phase plates [14,15], holograms [16], mode conversion techniques [17], axicon lenses [18,19], and hollow fibers [20]. Freeform Fresnel optics has recently emerged as a promising class of optical components. It combines the advanced wavefront control of freeform optics with the compact form factor of Fresnel structures. Goldstein proposed a design method for faceted freeform Fresnel lenses capable of producing prescribed output intensity distributions [21]. With the steady development of freeform Fresnel lens fabrication processes [22,23,24], they have been applied to compact wireless communication systems [25], irradiance shaping [26,27], and head-up displays [28].

In this study, we propose a method to generate a rectangular dark hollow beam using a freeform Fresnel lens. The lens aperture is divided into multiple fan-shaped sectors, each consisting of serrated annular Fresnel zones. A topological mapping is established between the sectorized sub-apertures and the target rectangular hollow pattern. This mapping allows each sector to redirect incident light to designated off-axis positions along the rectangular boundary. The lens is fabricated by ultra-precision diamond turning with a slow tool servo technique. Experimental results demonstrate that the generated beam profile is in good agreement with the simulations, validating the proposed method as a compact and manufacturable solution for structured beam generation.

## 2. Theoretical Design Method

**Figure 1:**
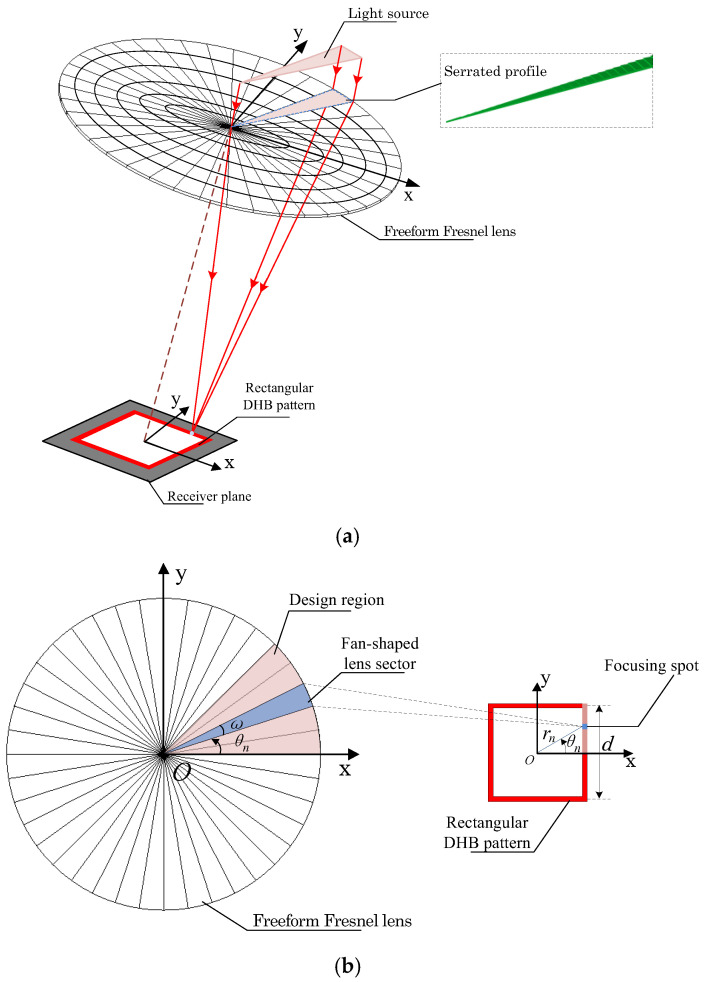
(**a**) Three-dimensional schematic diagram of the freeform Fresnel lens generating a rectangular DHB. (**b**) Topological mapping from fan-shaped lens sector to the receiver plane.

The fundamental principle for generating a rectangular DHB using a freeform Fresnel lens is illustrated in Figure 1a. The lens is divided into several sector-shaped regions with its center as the origin. Each sector generates an off-axis focal spot at a prescribed spatial location, and each sector consists of serrated annular zones. The superposition of these off-axis focal spots forms a rectangular intensity distribution on the detection plane. The topological mapping from the fan-shaped lens sectors to the target plane is shown in Figure 1b. A freeform Fresnel lens with a division angle of ω converts the normally incident light into a rectangular pattern with a side length of d. Light incident on a sector at an azimuth angle θ is mapped to a point on the rectangular pattern with the corresponding azimuth angle.

The rectangular pattern is both center-symmetric and diagonally symmetric. Therefore, the design range can be restricted to azimuth angles from 0 to π/4. If the design region is divided into n fan-shaped sectors, the angle ω of each sector is π/(4n). The distance $r_{n}$ from the focal point generated by the *n*th fan-shaped lens sector to the origin is given by

(1) $$r_{n}=\frac{d}{2\cos\left(\theta_{n}\right)}$$

where $\theta_{n}$ denotes the azimuth angle of the nth fan-shaped lens sector, given by

(2) $$\theta_{n}=\left(n-1\right)\omega$$

**Figure 2:**
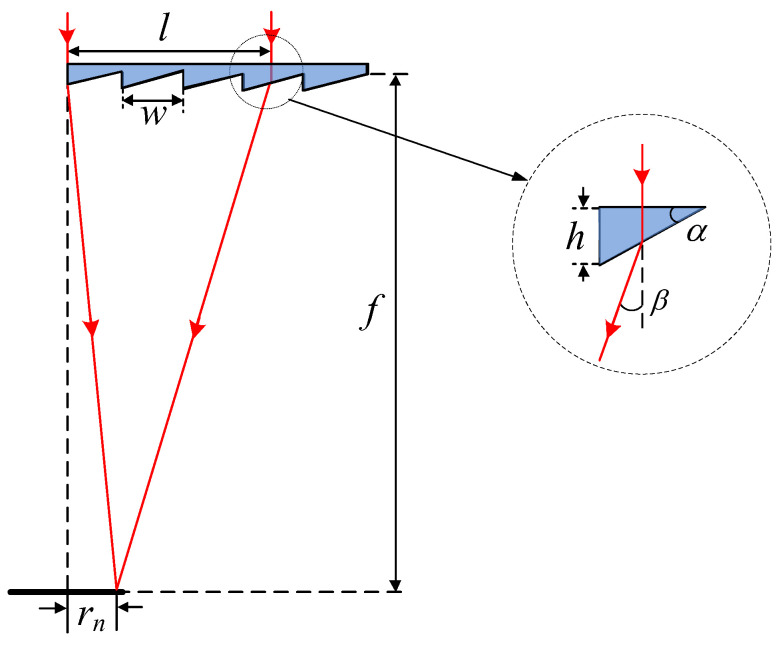
2D ray diagram of light incident on the lens facets.

A 2D ray diagram of light incident on the lens facets is shown in Figure 2. The freeform Fresnel lens consists of planar and groove surfaces, with the groove surfaces facing the receiver. A collimated incident light beam is refracted by the groove facet with a slope angle of α underneath the lens plane. For the *n*th fan-shaped lens sector, the facet angle α is given as

(3) $$\tan\left(\alpha \right)=\frac{N_{1}\sin(\beta )}{N_{2}-N_{1}cos(\beta )}$$

where

(4) $$\beta =\arctan\left[(l-r_{n})/f\right]$$

Here, $N_{1}$, $N_{2}$ are representative refractive index of the air and the Fresnel lens material respectively, l is the distance from the center of the facet to the center axis, and *f* is the focal length. If we denote the groove width of Fresnel lens as w, the height of the facet h can therefore be defined as follows:

(5) h=w×tan(α)

Monte Carlo ray-tracing simulations were performed using software TracePro ^®^ 7.0 (Lambda Research Corporation, Littleton, MA, USA) to evaluate the optical performance of the modular Fresnel lenses. The key parameters of the proposed freeform Fresnel lens are as follows: lens aperture D=25 mm, focal length f=125 mm, two grooves per millimeter (w=0.5 mm), groove height h<0.3 mm, material polymethyl methacrylate (PMMA, n=1.49,T=92.4% @ 640 nm), and side length of the rectangular DHB d=10 mm. A collimated light source with an output power of 1 W and a wavelength of 640 nm was used for simulation. The number of simulated rays is 500,000, and the receiving surface is a square plane with a side length of 15 mm and grid size of 128 × 128.

**Figure 3:**
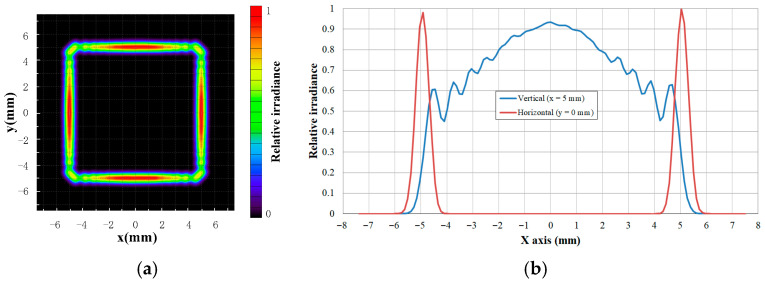
Relative irradiance distribution of the rectangular DHB for division angle of 5°. (**a**) Two-dimensional irradiance distribution. (**b**) Cross-sectional irradiance distribution curve.

Figure 3a shows the two-dimensional relative irradiance distribution of the rectangular DHB for a division angle of 5° (Total 72 sectors over the lens region). The simulation indicates that a square hollow spot with a side length of 10 mm and a hollow width of approximately 1 mm can be formed on the receiver plane. The irradiance distribution exhibits higher intensity along the central regions of each side, with noticeable stitching artifacts of focused spots at the corners. Figure 3b presents the cross-sectional irradiance profiles along x=5 mm and y=0 mm. The results confirm the absence of stray light within the hollow region. The minimum irradiance within the effective spot area is about 50% lower than the maximum intensity.

**Figure 4:**
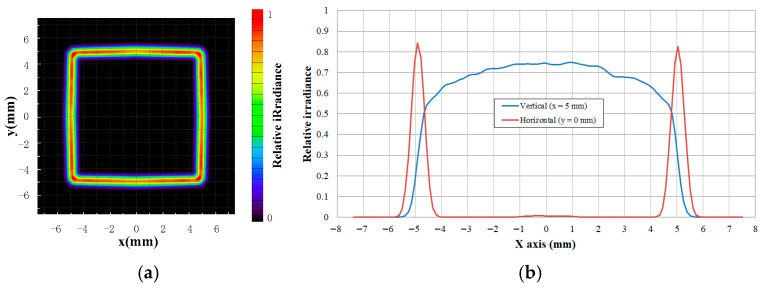
Relative irradiance distribution of the rectangular DHB for division angle of 2.5°. (**a**) Two-dimensional irradiance distribution. (**b**) Cross-sectional irradiance distribution curve.

The design principle of the proposed method is to focus the light from each fan-shaped sector into an individual spot, such that a linear intensity distribution is formed by superposing these focal spots. Owing to the finite angular span of each sector, the focal spot deviates from an ideal point and instead exhibits an arc-like profile. Accordingly, the precision of the angular division between sectors directly influences the beam intensity distribution on the receiver plane. To enhance the irradiance distribution uniformity of the rectangular DHB, the segmentation accuracy was increased to 2.5°. Figure 4 shows the corresponding relative irradiance distribution on the receiver plane. As seen in the figure, the intensity along the edges of the spot is significantly improved, leading to a more uniform rectangular pattern. Although reducing the division angle improves irradiance uniformity, it simultaneously increases machining complexity by narrowing the spacing between adjacent fan-shaped regions.

**Figure 5:**
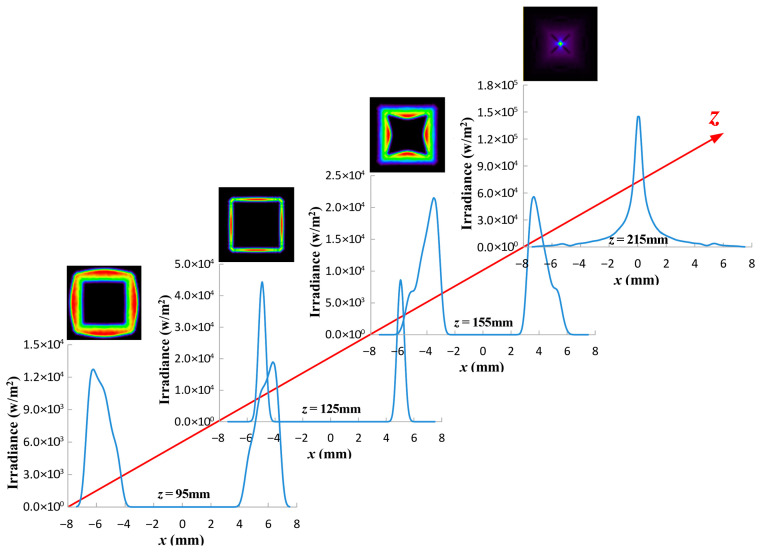
Cross line (y=0 mm) of the irradiance distribution of a rectangular DHB at various propagation distances of 5°.

The cross-sectional profiles of the irradiance distribution of the rectangular DHB in free space were calculated at various propagation distances z for a division angle of 5°, as shown in Figure 5. As the propagation distance increases, the hollow region of the optical spot gradually expands, reaching its designed maximum at the focal plane. At this position, the beam is most concentrated and its width is minimal. The hollow region gradually decreases beyond the focal plane. Although a focused spot distribution appears at the rear focal plane (z=215 mm), the beam as a whole has already diverged.

## 3. Experimental Verification

### 3.1. Experimental Setup

**Figure 6:**
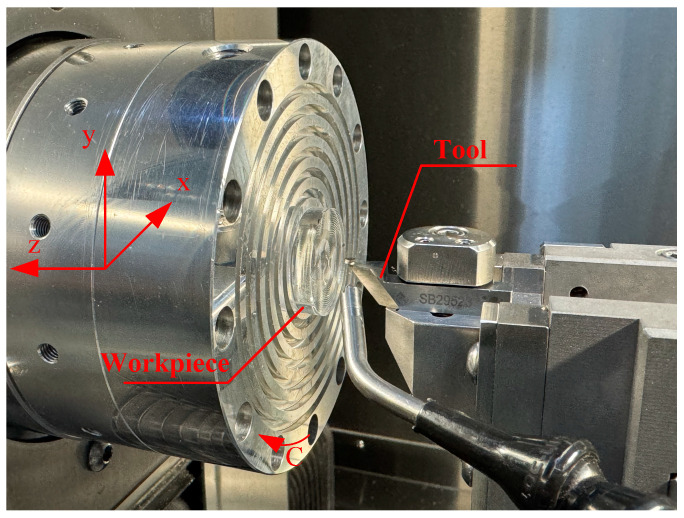
Experimental setup and coordinate configuration.

To validate the feasibility of the proposed method, a freeform Fresnel lens with a division angle of 5° was chosen for experimental fabrication, as it involves less machining complexity. The experiments were conducted on an ultra-precision lathe (Nanotech 650FG, Moore, Swanzey, NH, USA) equipped with three linear axes (X, Y, Z) and a rotational axis (C), as shown in Figure 6. A commercially manufactured single-crystal diamond tool was employed, with a rake angle of 0°, a nose radius of 10 µm, and an included angle of 70°.

### 3.2. Verification of the Exemplary Freeform Fresnel Optics

**Figure 7:**
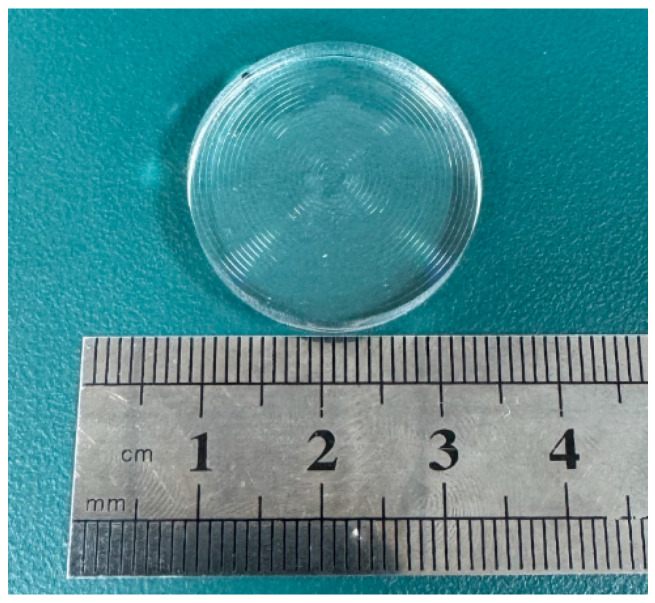
Photograph of the fabricated freeform Fresnel lens.

**Figure 8:**
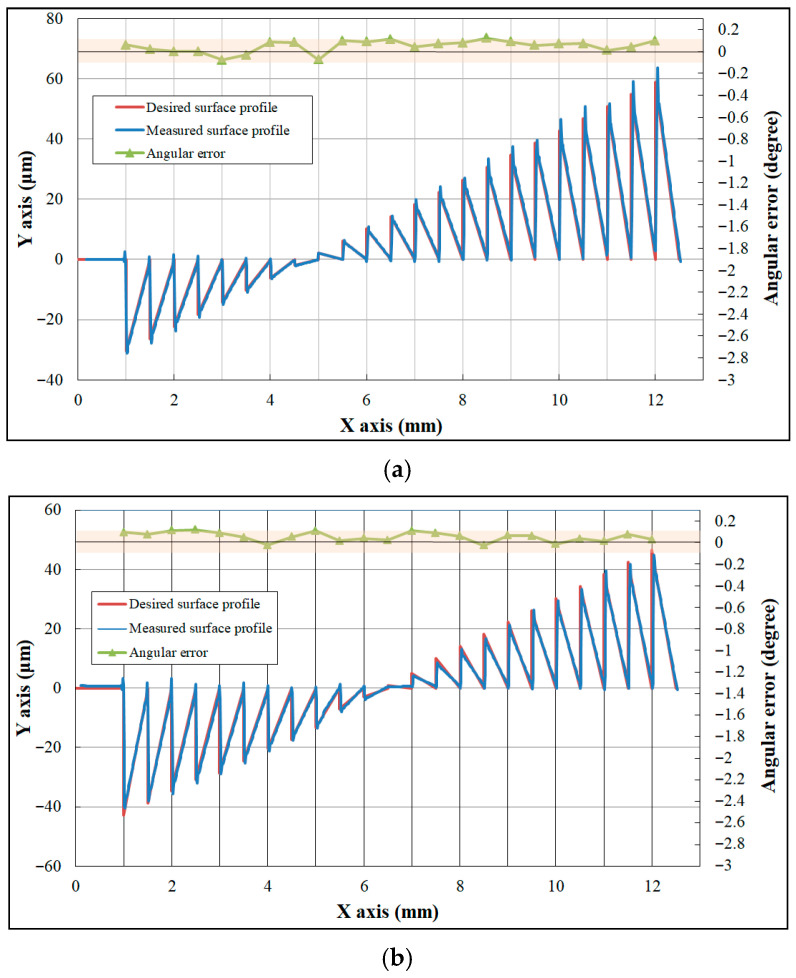
Comparison of the desired profile and measured profile of the freeform Fresnel optics on depth direction and corresponding forming error: (**a**) azimuth angles of 0°, (**b**) azimuth angles of 45°.

The freeform Fresnel lens with a division angle of 5°, introduced in Section 2, was successfully fabricated using ultra-precision machining, as shown in Figure 7. Due to the relatively small facet angles in the central region of the designed freeform Fresnel lens, the central area with a diameter of 2 mm was fabricated as a planar surface. A cross-sectional profile comprising 23 facets was measured using a probe-type optical instrument (Form Talysurf PGI, Taylor Hobson, Leicester, UK). Two line segments at azimuth angles of 0° and 45° were selected for analysis, with the testing starting points set at the central origin of the optical element (the X-axis of the ultra-precision lathe as the reference axis). The resulting contour lines, presented in Figure 8, were compared with the intended design, and the forming angular error was calculated. During measurement of the groove tip morphology, interference between the probe and the workpiece caused noise in the tip region. Therefore, only the valid data region was used for analysis. The maximum forming angular error was within 0.1°, demonstrating that the proposed fabrication process achieved high machining accuracy.

**Figure 9:**
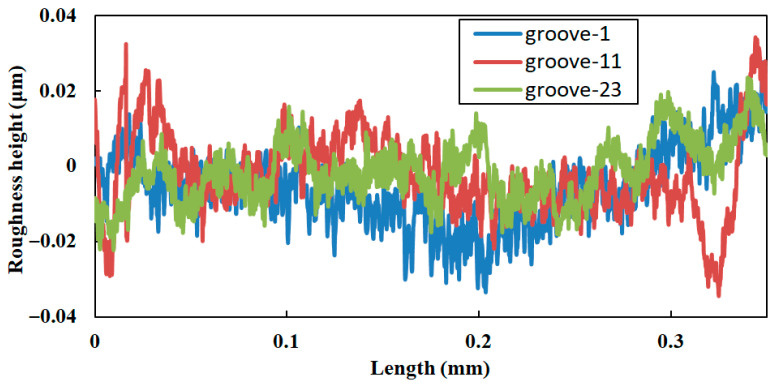
Measurement of surface roughness.

Based on the surface profile measurement results shown in Figure 8a, roughness analysis was performed on three grooves. Figure 9 presents the roughness curves of the inclined surfaces for the 1st, 11th, and 23rd grooves. The results indicate that the surface roughness values of the three groove inclined surfaces are 8.9 nm, 9.5 nm, and 8.2 nm, respectively. The experimental findings reveal that the surface roughness of groove-inclined facets is strictly confined to the nanometer scale, indicating excellent machining consistency and superior surface finishing quality.

**Figure 10:**
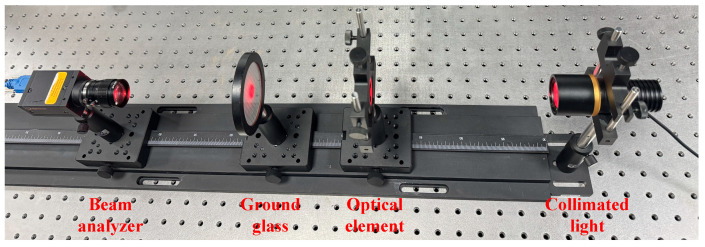
Photograph of the optical test setup.

A freeform Fresnel lens with division angle of 5°, fabricated from PMMA material, was processed to assess its optical performance, and an optical test setup was established as shown in Figure 10. The experimental test setup was assembled on a vibration-isolated optical platform, with all auxiliary testing components mounted on a graduated optical linear rail to enable precise positional alignment throughout the measurement process. A 640 nm laser integrated with a collimating lens was employed as the illumination source, which irradiated the machined freeform Fresnel lens; the transmitted beam was then imaged onto a receiving surface, and the corresponding light spot on this surface was subsequently captured by a beam analyzer (SP300, Ophir-Spiricon, North Logan, UT, USA). The freeform Fresnel lens was firmly fixed on a precision lens mount, with its grooved surface facing the receiving surface. The receiver employed herein is a ground glass slide, featuring a light-scattering surface on one side and a flat transparent surface on the other. The light spot was projected onto the scattering surface of the ground glass, while the beam analyzer acquired the spot image through its transparent flat side. This beam analyzer is equipped with a 7.1 mm × 5.3 mm CCD detector and a 5× magnification focusing lens.

**Figure 11:**
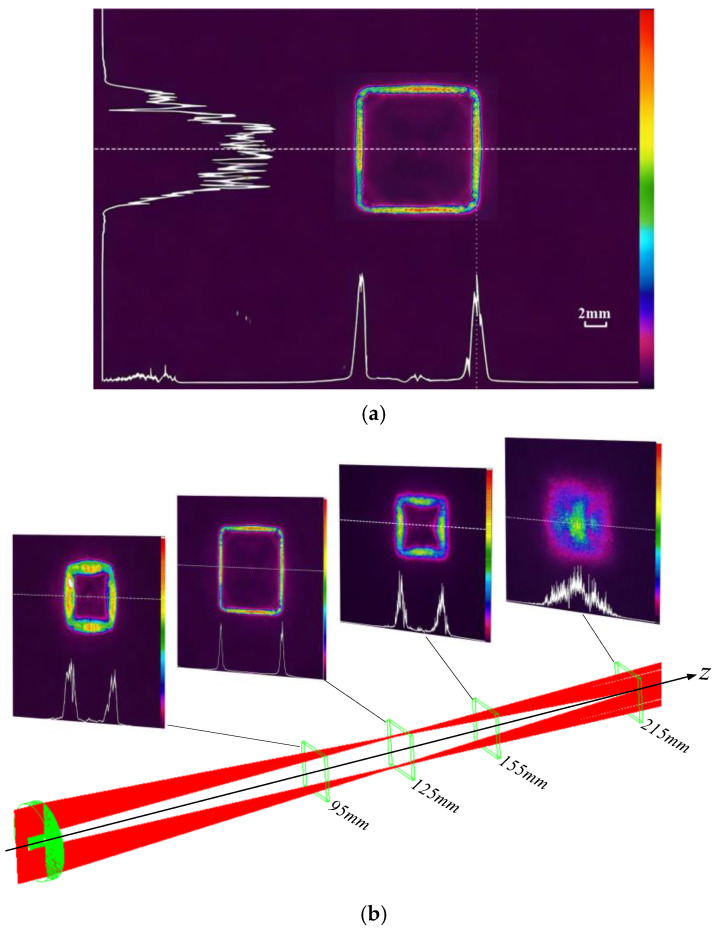
Optical performance test. (**a**) Measured beam profile test image at focal plane. (**b**) Cross-sectional optical path of beam propagation and spot distributions at different transmission positions.

Figure 11 presents the experimentally measured optical performance of the fabricated freeform Fresnel lens. As shown in Figure 11a, a rectangular dark hollow beam with a side length of 10.3 mm and a width of approximately 1.2 mm was obtained at the focal plane, where the central intensity is significantly suppressed and the boundary exhibits a continuous closed high-intensity distribution. The overall beam profile shows good agreement with the simulated result (Figure 3a), demonstrating that the proposed design method can effectively achieve the desired beam shaping after fabrication. Meanwhile, noticeable intensity of non-uniformity is observed along the boundary, particularly near the rectangular corners. This behavior is consistent with the simulation results, where the non-uniform energy distribution is attributed to the finite division angle accuracy of the fan-shaped sectors. Figure 11b further illustrates the evolution of the beam at different propagation distances. As the propagation distance increases from 95 mm to 125 mm (focal plane), the hollow region gradually expands and stabilizes, and the rectangular profile becomes more distinct. At the focal plane, the beam exhibits the most concentrated size and highest contrast. With further propagation to 155 mm and 215 mm, the beam begins to diverge, the contrast of the hollow region decreases, and the boundary becomes blurred, indicating a strong focal-plane dependence of the structure.

## 4. Conclusions

This study presents a design method for freeform Fresnel lenses to generate rectangular dark hollow beams. The approach is validated through theoretical modeling, ray-tracing simulations, and experimental characterization. The proposed method divides the lens into multiple fan-shaped sectors and establishes a topological mapping between each sector and the target rectangular hollow pattern. By redirecting light to off-axis positions through serrated Fresnel zones, the desired beam profile is formed via spot superposition. A freeform Fresnel lens with a division angle of 5° was successfully designed and fabricated. The experimental results agree well with the simulations, confirming the feasibility and effectiveness of the proposed method. Although reducing the division angle can improve irradiance uniformity, it also increases machining complexity. Therefore, a trade-off between optical performance and fabrication difficulty must be considered. The proposed design strategy is not limited to rectangular beams and can be extended to hollow beams with arbitrary geometries.

A concentric design with a constant groove width was adopted in this work, and when combined with the ultra-precision slow tool servo machining process, it effectively reduces machining difficulty and enables the convenient and efficient fabrication of the required surface profile. The fabricated lens exhibits an angular error below 0.1° and a surface roughness below 10 nm, demonstrating excellent machining performance.

Freeform Fresnel lenses replace continuous surfaces with segmented facets, significantly reducing thickness and weight. Although this may slightly compromise surface continuity, it enables lightweight and compact optical designs. Furthermore, such structures are generally considered compatible with fabrication techniques such as injection molding and nanoimprint lithography, indicating their potential for large-scale production. However, this aspect was not experimentally investigated in the present study and will be addressed in future work.

## Data Availability

The original contributions presented in this study are included in the article. Further inquiries can be directed to the corresponding author.
